# Metronomic Chemotherapy: Anti-Tumor Pathways and Combination with Immune Checkpoint Inhibitors

**DOI:** 10.3390/cancers15092471

**Published:** 2023-04-26

**Authors:** Elena Muraro, Lorenzo Vinante, Elisabetta Fratta, Alessandra Bearz, Daniela Höfler, Agostino Steffan, Lorena Baboci

**Affiliations:** 1 Immunopathology and Cancer Biomarkers Unit, Centro di Riferimento Oncologico di Aviano (CRO), IRCCS, 33081 Aviano, Italy; emuraro@cro.it (E.M.); efratta@cro.it (E.F.); asteffan@cro.it (A.S.); 2 Radiation Oncology Department, Centro di Riferimento Oncologico di Aviano (CRO), IRCCS, 33081 Aviano, Italy; lorenzo.vinante@cro.it; 3 Medical Oncology Department, Centro di Riferimento Oncologico di Aviano (CRO), IRCCS, 33081 Aviano, Italy; alessandra.bearz@cro.it; 4 Infections and Cancer Epidemiology, German Cancer Research Center (DKFZ), 69120 Heidelberg, Germany; d.hoefler@dkfz-heidelberg.de

**Keywords:** immune checkpoint, immunomodulation, metronomic chemotherapy

## Abstract

**Simple Summary:**

Metronomic chemotherapy, a continuous administration of a lowered dose of drugs without long breaks, is currently considered an alternative approach for the treatment of cancer patients experiencing drug resistance and/or toxic side effects. This therapy can lead to tumor control by inhibiting tumor angiogenesis, suppressing tumor cell growth, and indirectly boosting the anti-tumor immune response. A synergic therapeutic role was observed after the combined administration of metronomic chemotherapy and immune checkpoint inhibitors, in both preclinical and clinical settings. However, the optimal drug combinations, sequence, and optimal concentration–time factors should be evaluated in representative preclinical models. Here, we report the current knowledge of the underlying mechanisms of action of metronomic chemotherapy and the potential therapeutic effect when administered in combination with the immune checkpoint inhibitors, at both preclinical and clinical levels.

**Abstract:**

Increasing evidence pinpoints metronomic chemotherapy, a frequent and low dose drug administration with no prolonged drug-free intervals, as a potential tool to fight certain types of cancers. The primary identified targets of metronomic chemotherapy were the tumor endothelial cells involved in angiogenesis. After this, metronomic chemotherapy has been shown to efficiently target the heterogeneous population of tumor cells and, more importantly, elicit the innate and adaptive immune system reverting the “cold” to ”hot” tumor immunologic phenotype. Although metronomic chemotherapy is primarily used in the context of a palliative setting, with the development of new immunotherapeutic drugs, a synergistic therapeutic role of the combined metronomic chemotherapy and immune checkpoint inhibitors has emerged at both the preclinical and clinical levels. However, some aspects, such as the dose and the most effective scheduling, still remain unknown and need further investigation. Here, we summarize what is currently known of the underlying anti-tumor effects of the metronomic chemotherapy, the importance of the optimal therapeutic dose and time-exposure, and the potential therapeutic effect of the combined administration of metronomic chemotherapy with checkpoint inhibitors in preclinical and clinical settings.

## 1. Introduction

At present, conventional chemotherapy represents the pillar of standard care for human cancer. This therapeutic approach makes use of the administration of the highest dose of a chemotherapeutic agent with relatively high drug-free intervals, additionally referred to as the maximal tolerated dose (MTD) protocol. This approach aims to completely eradicate cancer cells targeting the cell cycle [1,2] and was shown to be successful in the clinical management of several types of human cancer [1,3]. Considering that most tumors are genetically heterogeneous and conventional chemotherapy treatment is cytotoxic prevalently to the therapeutic-sensitive tumor cell fraction, this treatment could drive the growth of therapeutic-resistant tumor cell clones between therapeutic cycles. This could be one of the explanations for tumor resistance and/or disease relapse observed for a fraction of cancer patients after conventional chemotherapeutic treatment [3].

In this context, metronomic chemotherapy (mCT), referred to as the continuous administration of a low dose of chemotherapeutic agents and minimal drug-free breaks [4,5], might represent an attractive alternative to conventional chemotherapy treatment. Although mCT was originally designed to overcome tumor resistance by targeting tumor endothelial cells [6], recent studies have shown that mCT could also activate anti-tumor innate and adaptive immunity as well as tumor cytotoxicity pathways [7]. On this ground, the combination of mCT with other pharmaceutical compounds, in particular the immune checkpoint inhibitors has recently been explored [8,9].

Of interest, mCT therapeutic benefits were observed in cancer patients where conventional chemotherapy was no longer effective. In particular, positive results were obtained in the management of metastatic breast cancer, non-small cell lung cancer (NSCLC), and colon rectal carcinoma after mCT administration [3]. Furthermore, the therapeutic combination of mCT with non-traditional cytotoxic agents, such as anti-angiogenic drugs, could effectively overcome host toxicity while preserving therapeutic efficacy [10]. Also, metronomic anti-tumor action was observed for some natural agents such as Sanguinarine, an alkaloidal agent able to hinder tumor metastasis, Cephalomannine, which inhibits cell viability, Reactive Oxygen Species production and migration in the hypoxic state of lung cancer cells, and Cordycepin, a natural compound that induces apoptosis and inhibits the epithelial-to-mesenchymal transition in oral squamous cell carcinoma cells [11,12,13].

Despite these findings, however, mCT treatment still primarily remains in the context of a palliative standard care tool rather than an upfront therapy. Therefore, further investigation is urgently needed to better refine the underlying mechanisms of the action of mCT and to identify the most effective chemotherapeutic drugs related to a tumor type concomitant with their optimal biological dose.

Here, we report the current knowledge and recent studies examining the anti-tumor mCT pathways with a particular focus on the potential therapeutic effect of mCT combined with immune checkpoint inhibitors.

## 2. Metronomic Chemotherapy Mechanisms of Action

**Figure 1:**
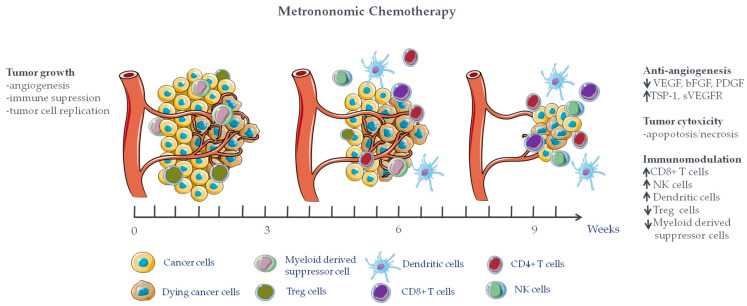
Schematic representation of the mechanisms of action of metronomic chemotherapy (mCT) on tumors. mCT is administered continuously using a low dose of chemotherapy for a period of time with no prolonged drug-free interruptions, resulting in effective inhibition of tumor angiogenesis and tumor growth and promoting the anti-tumor immune response. VEGFR, vascular endothelial growth factor; bFGF, basic fibroblast growth factors; PDGF, platelet-derived growth factor; TSP-1, Thrombospondin-1; sVEGFR, soluble vascular endothelial growth factor receptor-1; NK cells, natural killer cells; Treg cell, T regulatory cells. This figure was generated using Servier Medical Art, provided by Servier, and licensed under a Creative Commons Attribution 3.0 unported license.

Growing evidence has shown the capacity of different chemotherapeutic agents administered with a low dose and continuous scheduling to inhibit angiogenesis, to elicit the activation of the immune system, and to suppress tumor growth (Figure 1).

### 2.1. Anti-Angiogenic Effect

Angiogenesis, which refers to the growth of new blood vessels from existing vasculature, can occur locally through the involvement of differentiated endothelial cells or systematically through the recruitment of bone marrow endothelial cells [14]. Compared to other tumor cells, tumor-associated endothelial cells are characterized by a relatively lower replication rate; therefore, conventional chemotherapy was found to induce weak or temporary endothelial damage [15,16].

In a study by Browder et al., mCT cyclophosphamide treatment inhibited angiogenesis in resistant endothelial cell lines three-fold more effectively compared to the conventional treatment schedule. It is probable that the therapeutic benefit after the conventional treatment with cyclophosphamide could not be observed due to repair activation mechanisms of the endothelial cells during the therapeutic rest period [4]. Interestingly, a number of studies demonstrated that even extremely low concentrations of mCT (picomolar and/or nanomolar) could affect the in vitro replication of endothelial cells [17,18,19]. For instance, Bocci et al. reported that the in vitro exposure of endothelial cells to different low dose anti-cancer agents increased the expression of thrombospondin-1 [17], a matricellular protein which inhibits angiogenesis through direct effects on endothelial cell migration [20]. These findings were further confirmed in vivo, since the levels of expression of thrombospondin-1 and other angiogenesis inhibitors decreased following mCT treatment in mice models bearing different tumor types [21].

Circulating endothelial cells trigger neovascularization and thus play a crucial role in tumor growth [21,22,23]. Notably, mCT reduced the number of circulating endothelial cells in tumor-bearing mice models, whereas exposure to conventional chemotherapy strongly mobilized circulating endothelial cells after the end of the cycle treatment [22].

The hypoxia factor (HIF)-1α [24] and the vascular endothelial growth factor (VEGF) are widely recognized as important pro-angiogenetic factors [25]. Generally, a hypoxic microenvironment stimulates the expression of VEGF and the VEGF receptor 1–2, in both normal and neoplastic cells through an increase in the transcription of HIF-1α [26]. In this context, decreased HIF-1α levels were observed in several types of tumors after mCT [24], and similar results were reported following mCT topotecan treatment in ovarian cell lines [27].

It has been observed that mCT could efficiently target the tumor’s dormant cells, which are tumor cells able to survive in a non-proliferating state, that could disseminate or remain inactive for months, years, or even decades before re-emerging again originating in metastasis [3,28,29]. Conversely, this dormant cell phenotype could be observed after the completion of anti-cancer treatment [30,31]. Therefore, for the purpose of angiogenic dormancy, mCT could be used after the completion of conventional chemotherapy [32].

### 2.2. Immunomodulating Effects of mCT

The metronomic schedules of different chemotherapeutics have been shown to induce immunostimulatory effects at different levels [33]. In particular, the immunogenicity of mCT can arise from on-target effects, i.e., directly on cancer cells, or from off-target effects involving immune cells responsible for anti-tumor-promoting functions [33].

Focusing on the on-target effects, mCT can favor the induction of immunogenic cell death (ICD) and/or increase the susceptibility of tumor cells to immune effectors. ICD is defined as a form of regulated cell death sufficient to activate an adaptive immune response in an immunocompetent syngeneic host [34]. The stimulation of the adaptive immunity depends on two main parameters: antigenicity and adjuvanticity. Antigenicity is dependent on mechanisms favoring an efficient recognition of tumor-associated antigens (TAA) by the host’s adaptive immunity [35,36], whereas adjuvanticity of ICD is permitted by the release or the exposure of danger signals, referred to as damage-associated molecular patterns (DAMPs), which recruit and induce the maturation of professional antigen-presenting cells (APC), as dendritic cells (DCs) [36,37]. Activated APC in the tumor microenvironment present TAA to immune effectors, eliciting a specific anti-tumor immune response.

In this context, several chemotherapeutics were found to increase the antigenicity and adjuvanticity of dying tumor cells. For instance, mitomycin C promoted ICD by reprogramming tumor cell metabolism in favor of oxidative phosphorylation. The resulting increased permeabilization of mitochondrial membranes caused the release of mitochondrial DNA in the cytoplasm, favoring the activation of DCs [38]. Other DNA-damaging chemotherapeutics such as anthracyclines (idarubicin and doxorubicin) are able to induce the exposure or the release of DAMPs inducing the surface translocation of calreticulin or the release of high-mobility group box 1 (HMGB1) in dying tumor cells, both signals inducing the recruitment and the activation of DCs [39,40]. Cyclophosphamide can favor the release of uric acid during tumor cell death, another DAMPs able to activate APC in the tumor microenvironment [41,42]. Recently, Choi et al. reported that a new formulation of oral metronomic oxaliplatin up-regulated ICD markers both in vitro and in vivo, thus enhancing tumor antigen uptake and the activation of DCs in tumor-draining lymph nodes [43]. In addition to the ability to induce ICD, some chemotherapeutic agents such as doxorubicin, paclitaxel, and cisplatin, could also increase the sensitivity of tumor cells to immune effectors such as cytotoxic T lymphocytes (CTLs), by up-regulating the mannose-6-phosphate receptors and improving the permeability to granzyme B produced by CTLs [44]. Therefore, TAA-specific CTLs not only recognized and killed TAA-expressing tumor cells, but also neighboring tumor cells not expressing those TAA [44]. Similarly, pemetrexed, sensitizes NSCLC not only to CTLs activity but also to natural killer (NK) cells [45]. Finally, in pancreatic cancer cell lines, low levels of gemcitabine could increase the expression of MICA/B on the cell surface, enhancing innate immune function against tumor cells [46].

Anti-tumor immunity could be stimulated by the activation of immune effectors, such as DCs, CTLs, NK cells, and M1-like macrophages, or the depletion of immunosuppressive cells, such as M2-like macrophages, myeloid-derived suppressor cells (MDSC), and regulatory T (Treg) cells [7,33].

DCs are probably the major target for chemo-immunomodulation [39]. Low doses of cyclophosphamide could restore myelopoiesis and reverse the tumor-induced DCs paralysis observed at a systemic level and in situ by mobilizing DCs progenitors from bone marrow to the tumor site [7,41]. At the tumor site, low doses of paclitaxel favored the phagocytosis of tumor antigens by DCs and enhanced their maturation, differently to high doses of the same drug that caused DCs apoptosis [47]. Additionally, non-cytotoxic doses of doxorubicin, methotrexate, and mitomycin C directly improved the ability of DCs to efficiently present tumor antigens to T-cells in vitro. This capability was favored by the up-regulation of several molecules on the DCs cell surface, such as CD80, CD86, CD40, and MHC class-II [48]. Notably, the up-regulation of CD40 counteracted the DCs-suppression induced by tumor progression by preventing tumor-induced DCs apoptosis [49]. Interestingly, the up-regulation of the above reported molecules on DCs surface, together with the DCs increased production of IL-1β, IL-6, and IL-12 was also observed following treatment with relatively low concentrations (0.1–1 µmol/L) of vinblastine and vinorelbine (VNR) [50,51]. Importantly, some chemotherapeutics such as 5-fluorouracil and oxaliplatin were able to down-regulate the expression of a number of immunosuppressive ligands, including PD-L1, on the DCs surface [52]. On the other hand, several chemotherapeutic agents used at very low concentrations, such as methotrexate, could further increase the ability of DCs to induce T cell proliferation through enhancing the DCs maturation and antigen presentation [53].

Direct effects on CTLs and NK cells have been described for few chemotherapeutic drugs and specific metronomic schedules or therapy combinations. In vitro studies demonstrated an enhanced T cell activation after pemetrexed treatment, due to an increased mitochondrial respiratory capacity which promoted CTLs’ cytotoxic effects and the expression of co-stimulatory receptors [54].

Improved tumor infiltration by CD4^+^ and CD8^+^ T cells was observed after a combined treatment with low doses of paclitaxel and DC vaccination in a lung carcinoma xenograft model [55]. Likewise, cyclophosphamide administered at a low dose increased the frequency of IFN-γ-secreting CTLs and T helper 1 cells and ameliorated the cytotoxic activity of NK cells in tumor-bearing mouse models and cancer patients [56]. Low doses of cyclophosphamide were also able to recruit NK cells and to improve their anti-tumor cytotoxic activity in in vivo tumor models [57]. Finally, metronomic doses of cyclophosphamide favored the recruitment and activation of anti-tumor M1-like macrophages in the tumor microenvironment [5,58].

The ability to revert the immunosuppressive milieu typical of a tumor microenvironment and to restore the host’s anti-tumor immunity represents a pivotal challenge in the oncology field. mCT can exert this effect by selectively inhibiting immunosuppressive cells such as MDSCs and regulatory T cells (Tregs) and/or repolarizing them to immunostimulating effectors. In fact, by using cancer cell models, Michels et al. demonstrated that ultra-low doses of paclitaxel could stimulate the differentiation of MDSCs into DCs in a TLR4-independent way [59]. Furthermore, a selective elimination of Gr-1^+^ CD11b^+^ MDSCs was induced by gemcitabine administered in minimal doses in mouse cancer models, leading to an improved activation of CD8^+^ T cells and NK cells along with an increased anti-tumor immunity [60]. This effect was further amplified when gemcitabine was combined with genetic immunization through an adenovirus expressing IFN-β [60]. Interestingly, the mitigation of MDSC-mediated immunosuppression was stronger at a low dose (50 mg/kg) than at a high dose (100 mg/kg), compared to higher levels [61]. In the clinical setting, similar results were reported for metronomic doses of capecitabine in glioblastoma patients, showing decreased levels of circulating MDSCs and a consequent improved cytotoxic immune infiltration in the tumor microenvironment [62].

At present, it is widely demonstrated that metronomic cyclophosphamide has a strong impact on Tregs abundance locally and systemically, both in mouse models and in cancer patients. In vivo cancer models showed a block in the Tregs renewal after low dose cyclophosphamide administration, in particular at 45 day intervals [39,63]. The selective Tregs depletion might not only rely on the anti-angiogenetic effect of cyclophosphamide [64], but also depend on the ability of this drug to impair the TGF-beta pathway [65], which is known to be a critical regulator of Tregs development, function, and homeostasis [66]. Several clinical trials reported Tregs depletion after metronomic cyclophosphamide treatment in advanced cancer patients [64,67,68], and some of them also suggested a cyclophosphamide-dependent suppression of the inhibitory functions of Tregs [67,68]. In particular, metronomic treatment decreased CTLA-4 expression in activated Foxp3^+^ Tregs, thus selectively inhibiting this major subset of Tregs [67]. Similar to cyclophosphamide, other chemotherapeutics could induce Tregs depletion when administrated at low doses. For instance, the alkylating agent temozolomide reduced the intratumor Tregs/CD4^+^ ratios in tumor-bearing mouse models [69], whereas gemcitabine limited Tregs accumulation in mice affected by pancreatic adenocarcinoma [70].

### 2.3. mCT Directly Targets Tumor (Stem) Cells

Even though mCT has always been considered a palliative standard care tool, an increasing number of studies have demonstrated the direct cytotoxic effect of mCT treatment on tumor cells [5,71]. For instance, Orlandi and colleagues showed that metronomic VNR induced a decreased proliferation rate in two human NSCLC cell lines, wild-type and epidermal growth factor receptor (EGFR) tyrosine kinase inhibitor (TKI) mutated. Differently, the same VNR dose conveyed as conventional MTD chemotherapy treatment was proven effective only in EGFR wild-type NSCLC cell line [72].

Of particular interest are the results of the mCT cytotoxic effect on cancer stem cells and stem-like tumor-initiating cells. Even though these types of cells represent only a minor subpopulation of the tumor mass, they play an important role in tumor recurrence and metastasis. For instance, a study conducted on human pancreatic tumor xenografts showed a reduction of cancer stem cell numbers after metronomic treatment with cyclophosphamide [73]. Similarly, Chan and colleagues indicated that in vivo treatment with mCT prevented the phenotypic switch of carcinoma cells in tumor stem cells and prolonged mice survival. On the contrary, the same treatment conveyed as conventional MTD chemotherapy triggered the phenotypic conversion of survived carcinoma cells into stem-like tumor cells [74].

A recent work by Bodarenko et al. explored the impact of conventional MTD therapy compared to mCT on tumor heterogeneity. It was shown that mCT in vitro treatment of NSCLC cell lines, either resistant or sensitive to cisplatin and patupilone, limited the proliferation rate of both NSCLC cell types and the overall growth of 2D and 3D co-cultures. In contrast, following conventional MTD therapy, growth inhibition was observed in the drug-sensitive cells, but not in resistant ones. Notably, the survival of a low number of chemo-sensitive cell lines after mCT treatment was sufficient to limit the growth rate of resistant cells [9]. Altogether, these findings highlight the potential therapeutic role of mCT in the management of tumor resistance.

mCT can target tumor cells by exerting different mechanisms, such as apoptosis, caspase-independent apoptosis [75], senescence [76,77], non-apoptotic cell death [78], and immunogenic cell death [36]. A study reported that in vitro and in vivo treatments of neuroblastoma tumors with low doses of metronomic actinomycin D, either alone or combined with a pan-caspase inhibitor, led to apoptosis-independent cell death [78]. Elsewhere it was reported that mCT could induce tumor senescence of prostatic cells causing single- or double-strand break damage after treatment with a low dose (25 nM) doxorubicin [79]. The senescence induction was seen additionally with low dose topotecan in both the neuroblastoma cell line and in neuroblastoma xenografts [76].

mCT might also indirectly affect tumor growth by altering the cancer cells metabolism. Along this line, a study by Fares et al. reported that the combination of metronomic paclitaxel and AKT inhibitor perifosine led to an increased overall survival of NSCLC mice-bearing tumors. The observed efficacy was likely due to the inhibition of the two main metabolic pathways, the glycolytic metabolism and/or oxidative phosphorylation [80]. Several preclinical studies showed that the long-term exposure of chemotherapeutics could induce chemotherapeutic-drug dependency to the tumor-cell, and thus, the chemotherapeutic withdrawal might induce tumor cell death [81,82]. Therefore, the introduction of long-term exposure in mCT treatment could increase the killing efficacy even for the most resistant cancer cells [81].

### 2.4. mCT Mechanism of Actions Depend on Dose and Time-Exposure

There is increasing evidence that drug dose and temporal administration protocol might affect the anti-tumor mCT pathways in a non-linear way. Therefore, the dose and time-exposure of a given anticancer agent can impact the overall outcome of cancer therapy, even with the same total amount of the administered drug [83,84].

Based on these considerations, different preclinical studies have investigated the impact of the mCT dose and time-exposure on tumor regression and the activation of immune anti-tumor responses. In a pioneer study by Raymond et al., long-term exposure (14 days) to 0.29 µM paclitaxel induced a cytotoxic effect 3-times stronger than short-term exposure (1 h) to the same dose in human cancer cells (i.e., ovarian, breast, and non-small cell lung cancer cells). This work highlighted the crucial role of time-exposure in paclitaxel toxicity in human cancer cells, suggesting that long-term exposure could improve the antitumor activity [84]. Similarly, in vitro topotecan long-term exposure showed a higher response rate to a clonogenic assay when compared to short-term exposure, suggesting that topotecan was more active in the long-term continuous exposure [85].

More recent studies suggest that immune anti-tumor response is also strongly affected by dose and time-exposure and that this pathway could represent the main actor for tumor regression. In fact, Chen et al. showed that a cyclophosphamide dose of 140 mg/kg on an intermittent 6-day schedule induced a potent activation of innate immune cells (macrophages and NK cell DCs) that led to tumor regression. Interestingly, when used in an extensive intermittent schedule (every 9 days or 12 days), the equal amount of cyclophosphamide markedly decreased the number of infiltrating NK cells and tumor re-growth after 24 days. Therefore, in order to elicit a sustained antitumor immune cell recruitment, metronomic drug treatment must be at a sufficiently high dose and well-spaced in time [86].

Of interest, a study by Wu et al. demonstrated that a fully immune-competent mouse implanted with a brain tumor and treated with cyclophosphamide in a six day repeating metronomic schedule activated a potent antitumor innate and adaptive immunity that promoted tumor ablation. In contrast, an increased frequency of metronomic administration from every six days to every three days, or daily, did not elicit the immune response [84]. Consistently, an immunomodulatory effect of low dose metronomic cyclophosphamide was demonstrated in the breast cancer model as well [87].

A metronomic dose is usually calculated as a subtoxic dose of the MTD (i.e., nearly 1/10th of MTD) of chemotherapy drugs. Notably, when the daily dose of the metronomic schedule was too low, the treatment could not suppress tumor growth, emphasizing the existence of a minimally effective dose [5,88,89]. However, a number of studies indicated that the optimal biological dose (OBD), which is the drug’s lowest dose with the highest efficacy and with absent or lowest toxicity, might be used as a novel parameter for mCT [90,91,92,93]. The OBD of four metronomic chemotherapy regimens (cyclophosphamide, vinblastine, VNR, and cisplatin) was investigated in tumor-bearing mouse models by Shaked et al. [90]. The OBD was determined for each of the used regimens, and the levels of viable circulating endothelial progenitor cells (CEPs) in blood samples were measured one week after treatment and compared with untreated control animals. The study revealed a significant dose-dependent decrease in viable CEPs, suggesting that the level of circulating CEPs might be a useful biomarker of antiangiogenic activity and could be adopted in clinical trials [22].

Mathematical models represent another innovative approach that potentially could define the optimal combination between drugs, dose, and scheduling, taking into account all of the components related to tumor biology [91,94]. For instance, Faivre et al.’ in silico model predicted that a temozolomide metronomic regimen was the best treatment regimen to induce tumor regression through its anti-angiogenic effects; notably, clinical data further confirmed this hypothesis [95]. When the same model was extended to metronomic VNR for lung cancer patients, in silico simulations suggested that an alternative dosing of VNR (60–30–60 mg) was well tolerated and more effective in respect to all the other metronomic regimens tested [96]. Elsewhere, it was reported that metronomic gemcitabine proved to achieve a significant reduction in tumor growth in a resistant model of human neuroblastoma xenograft model, whereas standard MTD therapy was totally ineffective [97,98].

Overall, these findings evidence that chemotherapy dose and time-exposure are both crucial for a successful anti-tumor therapy. In addition, since the optimal biological dose and the precise schedule may vary between tumor models, they still remain challenging tasks to refine.

## 3. Synergistic Role of mCT with Immune Checkpoint Inhibitors

Due to its pleiotropic immunomodulatory activities, mCT represents an ideal partner to combine with immune checkpoint inhibitors (ICI) [7]. In recent years, immunotherapy with ICI was shown to boost the existing anti-tumor immune response by modulating the immune cell activation and cytotoxic activity [7,39]. So far, the most studied ICI targets are the T cell surface receptors, the cytotoxic T lymphocyte antigen-4 (CTLA-4), and the programmed cell death (PD-1) along with the corresponding PD-L1 or PD-L2 ligands [7]. In the clinical context, outstanding results were observed for several tumors, in particular for melanoma [99,100], lung cancer [101], and hematological malignancies [102]. Despite the promising developments in cancer immunotherapy, the successes of immune checkpoint blockade antibodies to treat various types of cancer are limited to a fraction of patients [80]. Hence, to avoid the immunological tumor escape and to increase the rate of ICI responders, the combination of immunotherapy with chemotherapy has been investigated for a wide variety of cancer types and has provided encouraging results, warranting further clinical investigations [103,104,105]. An overview of mCT and ICI treatment combinations tested in vitro and in vivo are listed in Table 1.

In this context, Khan et al. assessed the anti-tumor response in triple-negative breast cancer (TNBC) by comparing the effectiveness of three different cyclophosphamide regimens. Results showed that treatment in murine orthotopic models with cyclophosphamide of 140 mg/kg every 6 days induced a higher suppression of tumor growth when compared to a conventional MTD dose or daily metronomic dose. Even though, it was observed that a single metronomic treatment with cyclophosphamide increased PD-L1 expression in a breast tumor (in vitro and in vivo), treatment with both cyclophosphamide and anti-PD-1 antibody, did not elicit an increased immunomodulatory effect. Despite this unexpected finding, the study highlighted the potential use of metronomic cyclophosphamide for breast cancer treatment [106]. Interestingly, a paper by He and colleagues on squamous cell lung carcinoma syngenic murine models showed that mCT exerted a pronounced antitumor effect when combined with subsequent anti-PD-1 monoclonal antibody (mAb) treatment. In contrast, the effects of MTD chemotherapy combined with anti-PD-1 mAb appeared to be more additive than synergic. Moreover, the study reported that macrophages, CD8^+^ T lymphocytes, and gut microbiota might play a pivotal role in mediating the antitumor synergistic effects. Overall, the study provided evidence for a novel strategy for the optimization of chemotherapy in SCLC patients [107]. A low dose of carboplatin was also reported to increase both CD8^+^ T-cell infiltration and PD-L1 expression in the lung cancer mouse model, hence potentiating the anti-tumor effect of PD-1 inhibitors without adverse effects [108].

Similarly, a triple combined treatment of metronomic doses of gemcitabine with checkpoint kinase 1 (CHK1) inhibitor and anti-PD-L1/anti-PD-1, significantly increased antitumorigenic CD8^+^ cytotoxic T cells, DCs, and M1 macrophage populations in the SCLC model. In contrast, immunosuppressive M2 macrophage and MDSCs were significantly reduced following the combining treatment [101]. Petrizzo et al. reported that the triple combinatorial approach of a multi-peptide vaccine, a multi-drug metronomic chemotherapy, and an anti-PD-1 efficiently potentiated the vaccine anti-tumor effect inhibiting tumor growth in 66.6% of melanoma-bearing mice [109]. Finally, by using NSCLC in vitro and in vivo models, Skavatsou et al. demonstrated that exposure to combined gemcitabine and anti PD-1 mAb up-regulated the expression of TSP-1 and VEGF-A, thus restricting tumor angiogenesis. The combined regimen also enhanced anti-tumor immunity by increasing the CD8^+^ T lymphocytes number and the release of pro-inflammatory cytokine, along with reduced Tregs percentages [110].

**Table 1:** Overview of the immunologic findings in preclinical context of the combinatorial use of metronomic chemotherapy and immune checkpoint inhibitors.

Refs.	Treatment Drugs mCT + ICI	Tumor Type	Immunologic Outcome	Efficacy
[106]	Cyclophosphamide + anti-PD-1	Triple negative breast cancer	No increase of PD-1 or CD4+/CD8+ T cell	Not reported
[47]	Cisplatin/docetaxel + anti-PD-1	Squamous cell lung carcinoma	Increased macrophage Increased TIL (CD45+, CD3+, CD8+)	Not reported
[108]	Carboplatin + anti-PD-1	Lung cancer	Increased PD-1 expression Increased CD8+ T cell infiltration	Reduced tumor growth No adverse effect (mice body weight)
[101]	Gemcitabine + anti-PD-L1	Squamous cell lung carcinoma	Increased M1-like macrophage Increased CD8+ T cell infiltration Decreased M2-like macrophage	Complete inhibition of tumor growth
[109]	Multipeptide vaccine + XX+ anti-PD-1	Melanoma	Decreased Treg Increased INF-γ	Complete tumor inhibition
[110]	Gemcitabine + anti-PD-1	Lung cancer	Increased CD8+ T cell Decreased IL-10 Decreased Treg	Milder leucopenia Highest antitumor efficacy

mCT, metronomic chemotherapy; ICI, immune checkpoint inhibitors; PD-1, programmed death protein-1; TIL, tumor infiltrating lymphocytes; INF-γ, interferon-gamma; Treg, T regulatory cell; IL, interleukin.

From the preclinical experience it has emerged that metronomic schedules were generally more immunostimulatory compared to traditional MTD chemotherapy [111,112]. Accordingly, several clinical trials investigating the possible therapeutic benefits of a combined therapy of mCT with ICI are ongoing or have been completed (Table 2).

The TONIC trial conducted by Voorwerk et al. evaluated the efficacy of nivolumab, an anti-PD-1 mAb, after short-term induction with mCT (cyclophosphamide or cisplatin or doxorubicin), or irradiation or no induction, in patients with metastatic or incurable, locally advanced TNBC [113]. Overall, the study reported an improved objective response rate (ORR) of 20%, with the majority of responses in the cisplatin (ORR 23%) and doxorubicin (ORR 35%) cohorts. In these cohorts, the improved response rate was associated with a significant increase of tumor-infiltrating T cells and an up-regulation of PD-1/PD-L1-related genes [113]. Similarly, in SCLC patients treated with both low dose albumin-paclitaxel and nivolumab, an up-regulation of CD8+ T cells has been observed. Additionally, the study provided evidence that the subsequential administration of immunotherapy 24 h after low dose chemotherapy might be an optimal timing for the treatment efficacy of SCLC [107]. Conversely, Vergnenegre and colleagues in their clinical trial (Table 2, NCT03801304) did not observe a clinical benefit in terms of efficacy and safety when investigating the synergic effect of metronomic VNR combined with atezolizumab, an anti-PD-L1 mAb, in patients with an advanced stage of NSCLC [111].

## 4. Conclusions, Remarks, and Future Directions

So far, mCT has been used as a palliative standard care tool rather than an upfront therapy. However, increasing evidence supports mCT as a potential therapeutic tool to fight several tumor types. In fact, mCT has been reported to efficiently inhibit anti-angiogenesis and to target tumor heterogeneity. In addition, its ability to convert an immune “cold” into a “hot” tumor makes the mCT a potential tool to use in cancer management. mCT was developed to overcome conventional chemotherapy drug resistance. It is possible that some tumor cells can develop drug resistance after a prolonged administration of mCT treatment, however the mCT multiple mechanisms of action could effectively counteract this effect through the anti-angiogenic activity, thus preserving the therapeutic efficacy.

Considering that it is very unlikely that a single metronomic regimen could have universal efficacy for any given tumor type, the full potential use of mCT needs further investigation. To date, little is known regarding the identification of the most effective chemotherapeutic drugs related to tumor type, the optimal biological dose (pharmacokinetics/pharmacodynamics) of each agent to be used alone or in combination, and the timing of drug administration.

Although published data showed that mCT might be operative at different drug concentrations, little is known on the pharmacokinetics (PK) and pharmacodynamics (PD) of mCT. The mCT PK/PD is not a miniature version of high-dose PK/PD; therefore, it could have different kinetics reflecting in a different free drug concentration circulating in the plasma. This addresses the question as to whether PK/PD parameters may be used as a biomarker to predict the mCT clinical outcome.

In this regard, the current biomarkers employed to identify those patients who could benefit from mCT are mainly clinical parameters related to the advanced stage of cancer, the lack of a response to conventional chemotherapy (relapsed or refractory tumor), and/or the absence of tolerance to high-dose chemotherapy due to a high toxicity profile. We hypothesize that patients’ selection for the combined treatment of mCT with ICIs could be based by using the circulating biomarkers showing a promising predictive and prognostic value for ICIs therapy alone.. In particular, we refer to the baseline level and the dynamic changes of the neutrophil-to-lymphocyte ratio (NLR), the platelet-to-lymphocyte ratio (PLR), and the percentage of NK cells, PD1^+^CD8^+^ T cells, and MDSCs.

Considering the infinite number of potential combinations of metronomic chemotherapy and/or targeted therapy and/or radiotherapy as well, when alternated with conventional MTD chemotherapy appropriate mathematical models could be the best way to select the best combinations, before application in pre-clinical and clinical trials. Finally, metronomic treatment still requires the identification of mCT-associated biomarkers which can be easily measured before and after therapy. Even more challenging, there remains the need to identify biomarkers that can predict durable benefits of mCT in cancer patients who do not respond to conventional therapies or who have a disease-related relapse. If possible, these patients should be evaluated for causative mechanisms in order to better refine mCT treatments.

## Figures and Tables

**Table 2:** Overview of the ongoing clinical trials investigating the combined metronomic chemotherapy and immune checkpoint inhibitors for advanced tumor treatment.

mCT	ICI	Study Title	Status	Phase	Clinical Trial n°
Cyclophosphamide	Ipilimumab	A Phase I Clinical Trial of Combined Cryotherapy and Intra-tumoral Immunotherapy with Autologous Immature Dendritic Cells in Men with Castration-Resistant Prostatic Cancer and Metastases to Lymph Nodes and/or Bone Pre- or Post-Chemotherapy	Completed	I	NCT02423928
Cyclophosphamide	Nivolumab/Ipilimumab	Autologous Dendritic Cells and Metronomic Cyclophosphamide in Combination with Checkpoint Blockade for Relapsed High-Grade Gliomas in Children and Adolescents	Recruiting	I	NCT03879512
Vinblastine Cyclophosphamide Capecitabine	Nivolumab	Nivolumab in Combination with Metronomic Chemotherapy in Pediatrics Refractory/Relapsing Solid Tumors	Recruiting	I and II	NCT03585465
Gemcitabine Doxorubicin Docetaxel	Nivolumab	GALLANT: Metronomic Gemcitabine, Doxorubicin, Docetaxel, and Nivolumab for Advanced Sarcoma	Recruiting	II	NCT04535713
Temozolomide	Nivolumab	Temozolomide + Nivolumab in MGMT Methylated Oesophagogastric Cancer (ELEVATE)	Recruiting	II	NCT04984733
Temozolomide	Nivolumab + Ipilimumab	A Longitudinal Assessment of Tumor Evolution in Patients with Brain Cancer.	Recruiting	I	NCT03425292
Cyclophosphamide	Pembrolizumab	Phase 2 Study of an Immune Therapy, DPX-Survivac with Low Dose Cyclophosphamide Administered with Pembrolizumab in Patients with Persistent or Recurrent/Refractory Diffuse Large B-Cell Lymphoma (DLBCL)	Active, not recruiting	II	NCT03349450
Vinorelbine	Atezolizumab	VinMetAtezo Study: Trial to Evaluate Safety and Efficacy of Vinorelbine with Metronomic Administration in Combination with Atezolizumab as Second-line Treatment for Patients with Stage IV NSCLC	Completed	II	NCT03801304
Decitabine	PD-1 Antibody (SHR-1210)	Combined Chemotherapy and PD-1 Antibody (SHR-1210) with or without Low dose Decitabine Priming for Relapsed or Refractory Primary Mediastinal Large B-cell Lymphoma (rrPMBCL): Two Stage, Phase I/II Trial	Unknown	I and II	NCT03346642
Gemcitabine	Nivolumab	Low dose Gemcitabine Combined with Nivolumab for Second-line and Above-line Treatment of Non-small Cell Lung Cancer Metastatic	Not yet recruiting	IV	NCT04331626
Cyclophosphamide	Pembrolizumab	CHEMOIMMUNE Study: Evaluation of Pembrolizumab in Lymphopenic Metastatic Breast Cancer Patients Treated with Metronomic Cyclophosphamide (Safety Run-in Phase)	Completed	II	EudraCT n. 2016-002736-33
Cyclophosphamide	Avelumab	CONFRONT Phase I-II Trial: Multimodality Immunotherapy with Avelumab, Short-Course Radiotherapy, and Cyclophosphamide in Patients with Relapsed/metastatic Head and Neck Cancer	Ongoing	I and II	EudraCT n. 2017-000353-39

mCT, metronomic chemotherapy; ICI, immune checkpoint inhibitors. Notes: cytotoxic T-lymphocyte-associated protein 4 (CTLA-4) inhibitor: ipilimumab; programmed death -1 (PD-1) protein inhibitors: pembrolizumab and nivolumab; programmed death-ligand (PD-L1) protein inhibitors: avelumab and atezolizumab.

## Data Availability

Not applicable.
